# Comparison of platelet reactivity between prasugrel and ticagrelor in patients with acute coronary syndrome: a meta-analysis

**DOI:** 10.1186/s12872-020-01603-0

**Published:** 2020-10-01

**Authors:** Mingxiang Wen, Yaqi Li, Xiang Qu, Yanyan Zhu, Lingfang Tian, Zhongqin Shen, Xiulin Yang, Xianqing Shi

**Affiliations:** 1 Intensive Care Unit, Guizhou Provincial People’s Hospital, No. 58 Zhongshan East Road, Nanming District, Guiyang, 550002 Guizhou China; 2 Emergency Department, Guizhou Provincial People’s Hospital, Guiyang, 550002 Guizhou China; 3 Radiology Department, Guizhou Provincial People’s Hospital, Guiyang, 550002 Guizhou China; 4 Endocrine Department, The First People’s Hospital of Guiyang, Guiyang, 550002 Guizhou China

**Keywords:** Ticagrelor, Prasugrel, Acute coronary syndrome, Meta-analysis

## Abstract

**Background:**

This meta-analysis aimed to compare the effects of prasugrel and ticagrelor on high (HTPR) and low on-treatment platelet reactivity (LTPR) in patients with acute coronary syndrome (ACS).

**Methods:**

Eligible studies were retrieved from PubMed, Embase, and the Cochrane Library. HTPR and LTPR were evaluated on the basis of the vasodilator-stimulated phosphoprotein platelet reactivity index (VASP-PRI) and P2Y12 reaction units (PRUs). HTPR and LTPR were analyzed using risk ratios (RRs) and their 95% confidence intervals (CIs). Weighted mean difference (WMD) and 95% CI were used to calculate the pooled effect size of platelet reactivity (PR).

**Results:**

Fourteen eligible studies were obtained, which included 2629 patients treated with ticagrelor (*n* = 1340) and prasugrel (*n* = 1289). The pooled results showed that the prasugrel-treated patients had higher platelet reactivity than the ticagrelor-treated patients (PRU: WMD = − 32.26; 95% CI: − 56.48 to − 8.76; *P* < 0.01; VASP-PRI: WMD = − 9.61; 95% CI: − 14.63 to − 4.60; *P* = 0.002). No significant difference in HTPR based on PRU was identified between the ticagrelor and prasugrel groups (*P* = 0.71), whereas a lower HTPR based on VASP-PRI was found in the ticagrelor-treated patients than in the prasugrel-treated patients (RR = 0.30; 95% CI: 0.12–0.75; *P* = 0.010). In addition, the results showed a lower LTPR was observed in the prasugrel group than in the ticagrelor group (RR = 1.40; 95% CI: 1.08–1.81; *P* = 0.01).

**Conclusions:**

Prasugrel might enable higher platelet reactivity than ticagrelor. Ticagrelor could lead to a decrease in HTPR and increase in LTPR. However, this result was only obtained in pooled observational studies. Several uncertainties such as the nondeterminancy of the effectiveness of ticagrelor estimated using VASP-PRI or the definition of HTPR (a high or modifiable risk factor) might have affected our results.

## Background

Acute coronary syndrome (ACS), a common but serious type of coronary artery disease [1], is characterized by primary atherosclerotic plaque rupture and secondary completely or partially occlusive thrombus that leads to ST-segment elevation myocardial infarction (STEMI), non-STEMI (NSTEMI), and unstable angina [2]. Percutaneous coronary intervention (PCI) is considered the preferred treatment for ACS to prevent thrombotic cardiovascular events [3].

Dual antiplatelet therapy with aspirin and a P2Y12 blocker is currently considered the primary treatment for ACS patients undergoing PCI [4]. Clopidogrel is the most common P2Y12 blocker used in dual antiplatelet therapies; however, its delayed action, susceptibility to genetic polymorphisms, and significant inter-individual response variability limit its clinical efficacy in patients with ACS [5]. The novel P2Y12 receptor antagonists, prasugrel and ticagrelor, are clinically superior to clopidogrel in patients with ACS who have undergone PCI [4, 6]. Several prospective clinical studies have compared the therapeutic effects of prasugrel and ticagrelor on platelet reactivity (PR) in patients with ACS [7–21]. Schüpke et al. [22] reported that the incidence of death, myocardial infarction, or stroke was significantly lower in prasugrel-treated patients with ACS than in ticagrelor-treated. In addition, Alexopoulos et al. [8] demonstrated that ticagrelor induced a significantly higher platelet inhibition than prasugrel in patients with ACS treated with PCI. These studies mainly focused on the efficacy of the two antiplatelet agents. A subsequent meta-analysis compared the two treatments on the basis of high on-treatment platelet reactivity (HTPR) and revealed that ticagrelor had a lower HTPR than prasugrel [23]. Although previous meta-analysis has reported the effect of ticagrelor and prasugrel on PR [24], the main purpose of their article is to investigate the effects of different detection methods on the inhibition of platelet response of the two drugs. However, the effects of prasugrel and ticagrelor on HTPR and low on-treatment platelet reactivity (LTPR) in patients with ACS have not been systematically reported. Therefore, an integrative meta-analysis of the published results is necessary.

In the present study, we compared the effect of both treatments on HTPR and LTPR in patients with ACS. The evaluation criteria for PR were based on the vasodilator-stimulated phosphoprotein PR index (VASP-PRI) and P2Y12 reaction units (PRUs).

## Methods

### Search strategy

This study was performed in accordance with the Preferred Reporting Items for Systematics reviews and Meta-Analysis (PRISMA) guidelines [25]. No review protocol for this meta-analysis was registered before the study was undertaken. PubMed, Embase, and the Cochrane Library were searched for all the studies comparing ticagrelor and prasugrel treatments in patients with ACS that were published through February 11, 2020. The following search string was used: “prasugrel” AND “ticagrelor” AND “acute coronary syndrome” OR “ACS.” The references of the acquired articles were manually searched to identify more potential studies. Only English language articles were included.

### Selection criteria

All the included articles met the following criteria: (1) included patients with ACS; (2) compared the therapeutic effects of prasugrel and ticagrelor; and (3) outcomes contained HTPR, LTPR, or PR. Notably, the main end point of this meta-analysis was PR, which was detected by VerifyNow-P2Y12 function assay or VASP test. According to the standards of the literature, PR was divided into PRU and PRI.

The exclusion criteria were as follows: (1) retrospective analysis, review article, conference abstract, or protocol; (2) duplicate search result; (3) therapeutic effects of prasugrel or ticagrelor compared with those of other drugs; (4) included healthy individuals; (5) required outcomes not reported; (6) drug utilization study only identified medical expenses; or (7) preclinical study.

### Data extraction and quality assessment

The data were extracted independently by two authors (Xiang Qu and Yanyan Zhu) and included the following information: first author name, publication date, study type, study period, patient age and sex, sample size, therapeutic strategy, clinical presentation, study duration, testing standard, definitions of HTPR and LTPR, and outcome indicators (HTPR, LTPR, and PR). Disagreements were resolved by discussion with the corresponding author (Mingxiang Wen). Furthermore, the article quality of randomized controlled trials (RCTs) was verified using the Cochrane risk of bias assessment tool, while non-randomized cohort studies were evaluated using the Newcastle-Ottawa Scale (NOS) [26].

### Statistical analysis

The statistical analysis was performed using the RevMan 5.3 software. For categorical data (HTPR and LTPR), risk ratios (RRs) and their 95% confidence intervals (CIs) were set as evaluation indexes. For continuous data (PR), the weighted mean difference (WMD) and its 95% CIs were used to calculate the pooled effect size. In addition, for multiple assessments of residual PR, we selected the data of the last measurement as the principal analysis. Owing to the large differences in clinical and methodological data in the included studies, a random effects model was applied to estimate the combined effect size. Cochran’s Q test and the *I*^2^ index were used to quantify the degree of heterogeneity [27]. Briefly, *P* values of < 0.05 and/or *I*^2^ values of > 50% were considered indicative of significant heterogeneity; otherwise, differences were considered non-significant. In addition, to investigate potential sources of heterogeneity, a subgroup analysis was performed in terms of study type (RCT or cohort), testing time (< 24 h after loading dose or 5–30 days after treatment initiation), and special population (patients with STEMI or diabetes). Finally, the publication bias of the included studies was detected using Egger’s test.

## Results

### Data retrieval

**Fig. 1:**
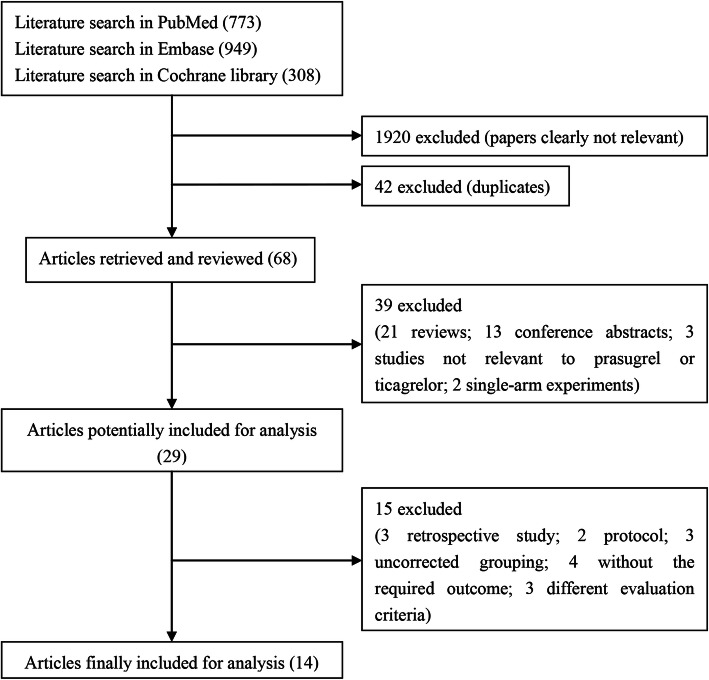
Flowchart of the data retrieval process

A flowchart of the literature search and selection process is shown in Fig. 1. A total of 2030 studies were identified from PubMed (773), Embase (949), and the Cochrane Library (308) in accordance with the initial search strategy. Of the studies, 1920 irrelevant studies and 42 repetitive studies were excluded. Among the remaining 68 studies, 39 were removed (details are shown in Fig. 1). Thereafter, 15 articles (three reviews, two protocols, three uncorrected groupings, four without interested outcomes, and one with different evaluation criteria) were excluded. Finally, 14 eligible studies were included in this meta-analysis [7–9, 11–19, 21, 28].

### Characteristics of the included studies

The detailed characteristics of the 14 included studies are summarized in Table 1. The publication dates of these studies ranged from 2012 to 2017. Among these studies, 10 were RCTs and four were prospective cohort studies, which included a total of 2629 patients with ACS treated with ticagrelor (*n* = 1340) or prasugrel (*n* = 1289).

**Table 1:** Characteristics of the included studies

Study	Study type	Study period	Population	No. of patients	Mean age (year)	Clinical presentation		Treatment		Testing time	Testing standard	Definition of HTPR	Definition of LTPR
						Ticagrelor	Prasugrel	Ticagrelor	Prasugrel				
**Alexopoulos 2014** [8]	cohrot study	NR	ACS undergoing PCI	T: 278 (232/46)	T: 60.6 ± 11.8	STEMI: 154	STEMI: 130	90 mg bid MD	10 mg MD	1 month post discharge	PRU	PRU > 208	NR
							NSTEMI: 63						
				P: 234 (200/34)	P: 58.4 ± 10.2	NSTEMI: 69	UA: 41						
						UA: 55							
**Alexopoulos 2012** [7]	RCT	NR	ACS and HTPR patients undergoing PCI	T: 22 (19/3)	T: 61.3 ± 8.1	STEMI: 8	STEMI: 11	90 mg bid, 15 days	10 mg QD, 15 days	15 days of treatment	PRU	PRU ≥ 235	NR
						NSTEMI: 7	NSTEMI: 3						
				P: 22 (18/4)	P: 58.3 ± 8.6		UA: 8						
						UA: 7							
**Alexopoulos 2013** [9]	RCT	2012.06–2012.09	ACS patients with DM	T: 15 (14/1)	T: 65.4 ± 7.7	STEMI: 3	STEMI: 4	90 mg bid, 15 days	10 mg QD, 15 days	15 days of treatment	PRU	PRU ≥ 230	NR
						NSTEMI: 7	NSTEMI: 4						
				P: 15 (14/1)	P: 60.9 ± 8.0		UA: 7						
						UA: 5							
**Deharo 2013** [11]	RCT	2013.03–2013.06	patients admitted for ACS	96 (78/18)	60.8 ± 9.8	NR	NR	90 mg bid	10 mg QD	1 month after ACS	PRI VASP	PRI VASP≥50%	PRI VASP ≤20%
				T: 48; P: 48									
**Deharo 2014** [12]	RCT	2013.03–2013.12	patients admitted for ACS	T: 93; P: 93	NR	NR	NR	90 mg bid	10 mg QD	1 month after ACS	PRI VASP	PRI VASP≥50%	PRI VASP ≤20%
**Dillinger 2014** [13]	cohrot study	NR	consecutive patients admitted for ACS	T: 119	59	NR	NR	NR	NR	during the hospitalization for ACS	PRI VASP	NR	VASP-PRI < 16%
				P: 268									
**Franchi 2016** [14]	RCT	2014.03–2015.10	underwent PCI in the setting of an ACS	T-M: 27 (20/7)	T-M: 57 ± 7	NR	NR	T-M: 90 mg bid MD	10 mg QD MD	1 week after randomization	PRU	PRU > 208, PRI > 50%	NR
					T: 54.8 ± 10.5						PRI-VASP		
				T: 25 (21/4)									
					P: 57 ± 6.9								
				P: 27 (21/6)									
**Guimaraes 2017** [28]	RCT	2013.07–2015.12	Patients with STEMI	T: 25 (18/7)	T: 52.2 ± 8.1	STEMI	STEMI	90 mg MD bid	60 mg LD	24 h after inclusion	PRU	NR	NR
					P: 55.5 ± 8.3								
				P: 25 (22/3)									
**Kerneis 2015** [15]	cohrot study	NR	Patients with STEMI underwent PCI	T: 58 (49/9)	T: 59.97 ± 1.54	STEMI	STEMI	90 mg MD bid	10 mg MD QD	30 d after primary PCI	PRU, VASP-PRI	VASP-PRI > 50%,	VASP-PRI < 16%
													PRU < 85
				P: 60 (49/11)	P: 57.78 ± 1.37							PRU > 208	
**Laine 2014** [16]	RCT	2012.10–2013.02	DM patients undergoing PCI for an ACS	T: 50 (33/17)	T: 64.8 ± 8.9	STEMI or NSTEMI: 41	STEMI/NSTEMI: 40	180 mg LD, 90 mg BID MD	60 mg LD, 10 mg QD MD	6–18 h post-LD	VASP	VASP> 50 and 61%	VASP< 16%
					P: 62.8 ± 8.2								
				P: 50 (43/7)			UA: 10						
						UA: 9							
**Laine 2015** [17]	RCT	2012.08–2013.06	STEMI patients with ongoing ischemia admitted for primary PCI	T: 44 (40/4)	T: 57.4 ± 9.8	STEMI	STEMI	180 mg LD	60 mg LD	6-12 h after the LD and before the first MD	VASP	VASP≥50%	VASP< 16%
					P: 54.7 ± 8.3								
				P: 44 (37/7)									
**lhermusier 2014** [18]	RCT	2012.11–2013.06	Patients admitted for ACS	T: 10 (10/0)	T:75(70–78)	NR	NR	90 mg bid	10 mg bid	24 h ± 4 after inclusion	PRU, PRI- VASP	PRU ≥ 208	NR
												PRI ≥50%	
				P: 10 (9/1)	P: 64 (52–68)								
**Parodi 2013** [19]	RCT	NR	STEMI patients undergoing primary PCI	T: 25 (19/6)	T: 67 ± 10	STEMI	STEMI	180 mg LD	60 mg LD	2 h after LD	PRU	PRU ≥240	NR
					P: 67 ± 14								
				P: 25 (20/5)									
**Yudi 2016** [21]	cohort study	2009.07–2013.11	consecutive ACS patients	T: 526 (411/115)	T: 61.7 ± 11.8	STEMI: 288	STEMI: 230	NR	NR	NR	NR	NR	NR
							NSTEMI: 138						
				P: 368 (317/51)	P: 57.1 ± 9.7	NSTEMI: 238							

Abbreviations: *RCT* randomized controlled trial, *ACS* acute coronary syndrome, *PCI* percutaneous coronary intervention, *DM* Diabetes Mellitus, *STEMI* ST segment elevation myocardial infarction, *NSTEMI* non ST segment elevation myocardial infarction, *UA* unstable angina, *CAD* coronary artery disease, *HTPR* high on-treatment platelet reactivity, *LTPR* low on-treatment platelet reactivity, *T* ticagrelor, *P* prasugrel, *bid* twice a day, *QD* once a day, *MD* maintenance dose, *LD* loading dose, *VASP-PRI* vasodilator-stimulated phosphoprotein platelet reactivity index, *PRU* P2Y12 reaction units, *NR* not reported

### Quality assessment

**Fig. 2:**
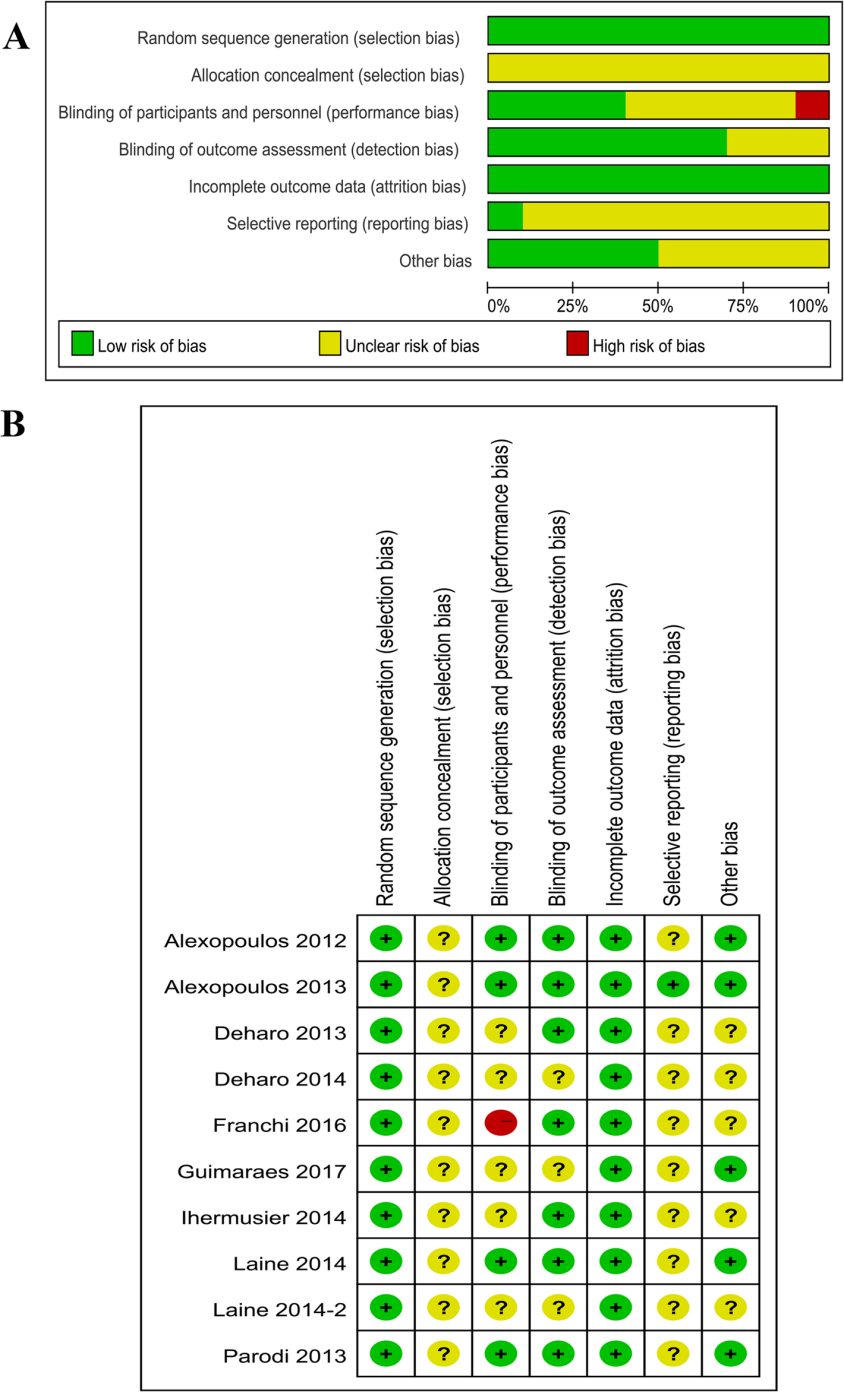
Quality assessment of the included studies. **a** Risk (%) of bias among the included studies. Green represents low risk of bias; yellow, unclear risk of bias; and red, high risk of bias. **b** Risk of bias items among the 10 included studies. +,?, and − indicate low, unclear, and high risk of bias, respectively

The results of the Cochrane risk of bias assessment showed that all 10 RCTs were of high quality (Fig. 2a) but did not describe the group allocation concealment process. The risk of bias was low for random sequence generation and blinding of outcome assessments in all 10 RCTs. Only in the study by Franchi et al. [14], the participants and researcher were not blinded (Fig. 2b). The NOS scores of the included cohort studies ranged from 5 to 8, implying that all were of high quality (Table 2).

**Table 2:** Quality assessment of the included cohort studies^a^

Cohort	Representativeness of the exposed cohort	Selection of the unexposed cohort	Ascertainment of exposure	Outcome of interest not present at start of study	Control for important factor or additional factor^b^	Outcome assessment	Follow-up long enough for outcomes to occur	Adequacy of follow-up of cohorts^c^	Total quality scores
Alexopoulos 2014 [8]	☆	☆	☆	☆	☆	☆	☆	☆	**8**
Dillinger 2014 [13]	☆	☆	☆	☆	☆	☆	☆	☆	**8**
Kerneis 2015 [15]	☆	☆	☆	–	☆	☆	☆	☆	**7**
Yudi 2016 [21]	☆	☆	☆	☆	–	☆	–	–	**5**

^a^ A study could be awarded a maximum of one star for each item except for the item Control for important factor or additional factor

^b^ A maximum of 2 stars could be awarded for this item. Studies that controlled for cardiovascular disease received one star, whereas studies that controlled for other important confounders such cancer received an additional star

^c^ A cohort study with a follow-up rate > 75% was assigned one star

### PR assessment

PR was evaluated on the basis of the PRU and VASP-PRI values. The PRU values were obtained using the VerifyNow P2Y12 assay (VN-P2Y12; Accumetrics Inc., San Diego, CA, USA), while the VASP values were evaluated using a commercially available kit (VASP; Biocytex, Marseille, France) and performed using a flow cytometer. Notably, the evaluation criteria in our analysis were as follows: PRUs of ≥208 or ≥ 230 and VASP-PRI values of > 50% were defined as HTPR, whereas VASP-PRI values of < 16% or PRUs of < 85 were defined as LTPR.

**Fig. 3:**
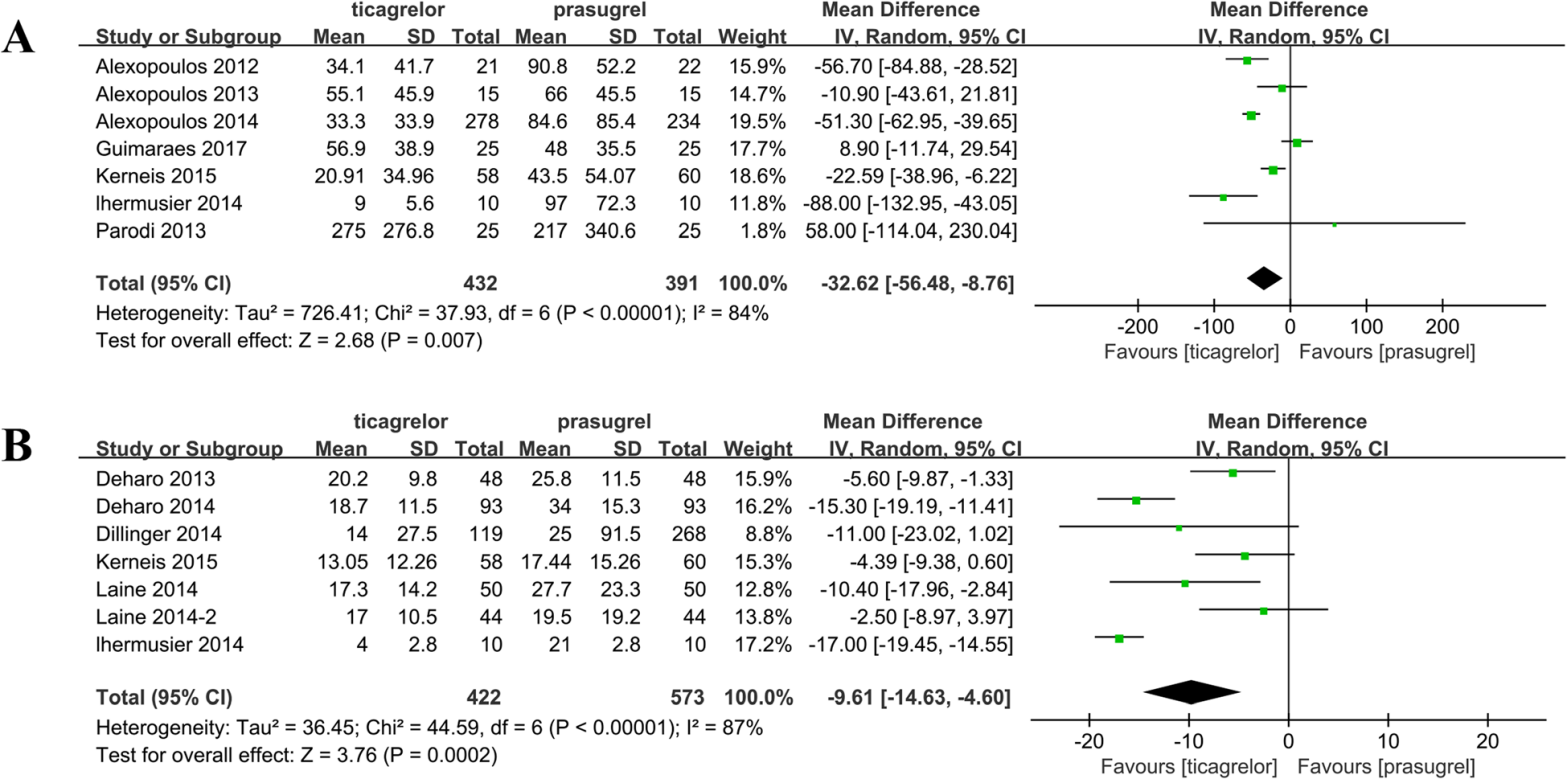
Results of the comparison of PR between the ticagrelor and prasugrel groups. **a** Forest plot of the comparison of PRU outcomes between the ticagrelor and prasugrel groups. **b** Forest plot of the comparison of VASP-PRI outcomes between the ticagrelor and prasugrel groups. PR, platelet reactivity; PRU, P2Y12 reaction unit; VASP-PRI, vasodilator-stimulated phosphoprotein platelet reactivity index

Among the included cohort studies, the pooled risk ratio from the combined data of the seven PRU studies showed that prasugrel treatment had a higher PR (WMD = − 32.62; 95% CI: − 56.48 to − 8.76; *P* < 0.01; Fig. 3a) than ticagrelor treatment, and heterogeneity was observed between studies (*P* < 0.01). Moreover, the pooled analysis of VASP-PRI indicated that the PR in the prasugrel group was significantly higher than that in the ticagrelor group (WMD = − 8.06; 95% CI: − 12.98 to − 3.14; *P* = 0.001; Fig. 3b). A significant heterogeneity was observed, with a *P* value of < 0.01 and *I*^2^ of 87%. Furthermore, to examine the sources of heterogeneity, a subgroup analysis was performed. In the PRU group, a subgroup analysis according to study type revealed that the PR in the prasugrel group was significantly higher than that in the ticagrelor group in the cohort studies (WMD = − 37.55; 95% CI: − 65.66 to − 9.44; *P* = 0.009). Meanwhile, significant differences were observed at 5–30 days after treatment initiation (WMD = − 36.72; 95% CI: − 57.04 to − 16.40; *P* < 0.01). In the PRI group, the PR in the prasugrel group was markedly higher than that in the ticagrelor group in the RCT and cohort. The included studies were classified according to testing time (< 24 h after the loading dose and 5–30 days after treatment initiation). The PR was high in the ACS patients with diabetes, which was treated with prasugrel (WMD = − 10.40; 95% CI: − 17.96 to − 2.84; *P* = 0.007; Table 3).

**Table 3:** Outcomes of the subgroup analysis

	No. of studies	Heterogeneity test		Effect size	
		I^2^ (%)	P_H_	WMD/RR (95% CI)	P
Platelet reactivity (PRU)					
Type of studies					
RCT	5	86	< 0.001	−29.93 (−70.21, 10.36)	0.15
Cohort	2	87	0.005	−37.55 (−65.66, −9.44)	0.009
Testing time					
< 24 h after the loading dose	3	87	0.005	−22.48 (−105.72, 60.76)	0.60
5–30 days after initiation of treatment	4	76	0.006	−36.72 (−57.04, −16.40)	< 0.001
Special Population					
STEMI	3	67	0.05	−5.77 (−34.85, −23.31)	0.70
Diabetic patients	1	–	–	− 10.90 (−43.61, 21.81)	0.514
Platelet reactivity (PRI)					
Type of studies					
RCT	5	88	< 0.001	−10.51 (− 16.17, −4.85)	< 0.001
Cohort	2	0	0.319	−5.36 (−9.97, −0.76)	0.023
Testing time					
< 24 h after the loading dose	3	89	< 0.001	−10.30 (−19.70, −0.90)	0.032
5–30 days after initiation of treatment	4	81	< 0.001	−8.91 (−15.03, −2.79)	0.004
Special Population					
STEMI	2	0	0.650	−3.69 (−7.63, 0.26)	0.067
Diabetic patients	1	–	–	−10.40 (−17.96, −2.84)	0.007
HTPR rates (PRU) ^a^					
Type of studies					
RCT	1	–	–	5.00 (0.25, 99.51)	0.29
Cohort	1	–	–	0.03 (0.00, 0.52)	0.016
Testing time					
5–30 days after initiation of treatment	2	84	0.01	0.39 (0.001, 62.72)	0.71
HTPR rates (PRI)					
Type of studies					
RCT	5	20	0.290	0.30 (0.12, 0.75)	0.010
Testing time					
< 24 h after the loading dose	2	0	0.440	0.31 (0.10, 0.98)	0.047
5–30 days after initiation of treatment	3	69	0.073	0.24 (0.03, 2.08)	0.197
Special Population					
STEMI	1	–	–	0.11 (0.01, 2.00)	0.137
Diabetic patients	1	–	–	0.38 (0.11, 1.33)	0.129
LTPR rates (PRI)					
Type of studies					
RCT	4	82	0.0008	1.63 (0.95, 2.80)	0.073
Cohort	2	85	0.009	1.31 (0.98, 1.76)	0.066
Testing time					
< 24 h after the loading dose	2	0	0.898	1.09 (0.86, 1.39)	0.462
5–30 days after initiation of treatment	4	89	< 0.001	1.69 (1.12, 2.56)	0.012
Special Population					
STEMI	2	0	0.634	1.15 (1.01, 1.30)	0.029
Diabetic patients	1	–	–	1.11 (0.79, 1.56)	0.545

^a^ three studies don’t involved in the results of STEMI or Diabetic patients

### HTPR and LTPR assessments

**Fig. 4:**
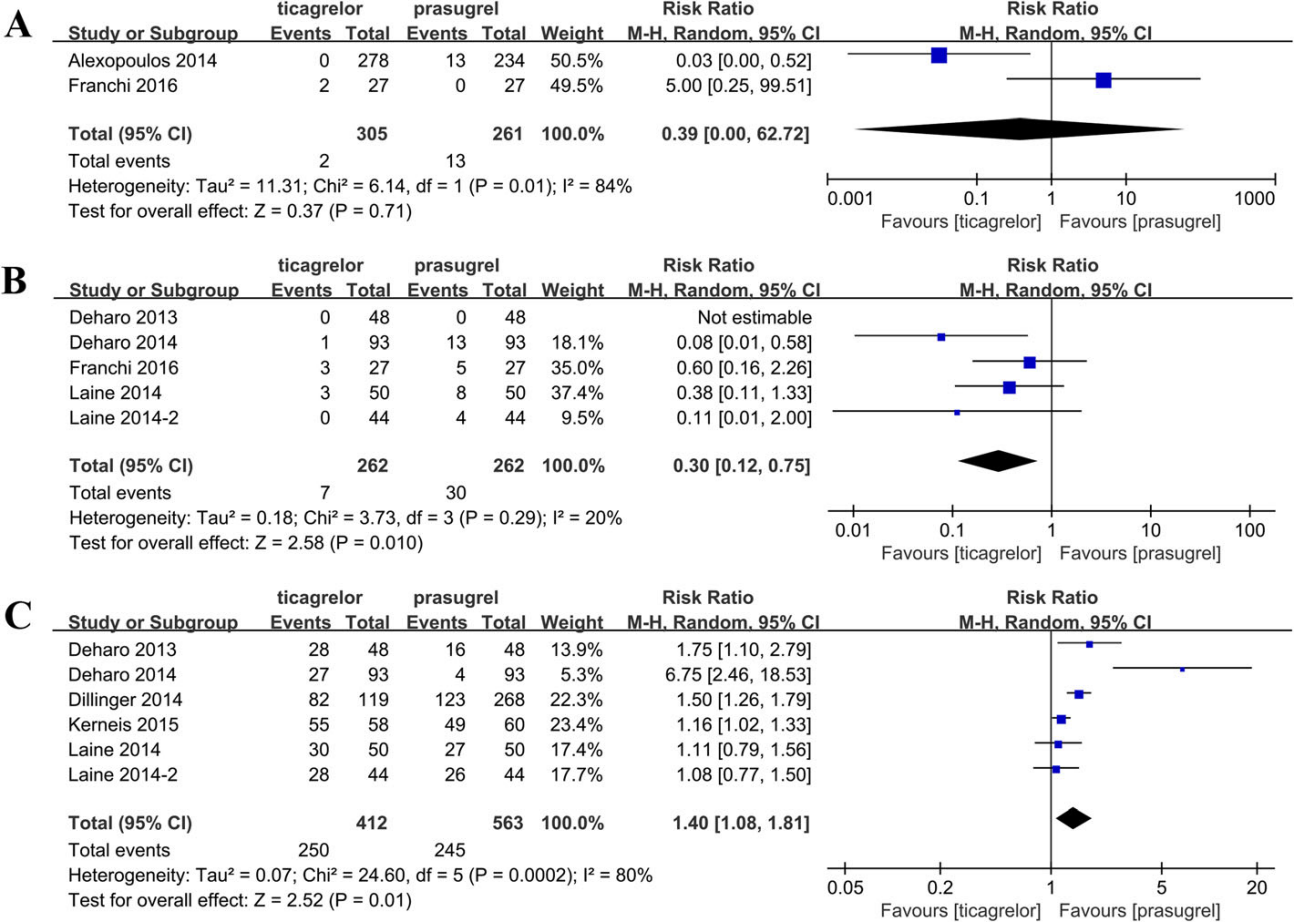
Results of the comparison of HTPR and LTPR between the ticagrelor and prasugrel groups. **a** Forest plot of the comparison of HTPR between the ticagrelor and prasugrel groups based on PRU and **b** VASP-PRI. **c** Forest plot of the comparison of LTPR between the ticagrelor and prasugrel groups based on VASP-PRI. HTPR, high on-treatment platelet reactivity; LTPR, low on-treatment platelet reactivity; PRU, P2Y12 reaction unit; VASP-PRI, vasodilator-stimulated phosphoprotein platelet reactivity index

Among the included studies, only two studies reported PRU-based HTPR. PRU-based HTPR was observed in 0.66% (2/305) of the ticagrelor-treated patients and 4.98% (13/261) of the prasugrel-treated patients. The pooled results revealed no significant difference between the prasugrel and ticagrelor groups (*P* = 0.71; Fig. 4a), and heterogeneity was observed between studies *(P* = 0.01, *I*^2^ = 84%). Moreover, five studies (all RCTs) reported HTPR based on VASP-PRI. PRI-based HTPR was observed in 2.67% (7/262) of the ticagrelor-treated patients and 11.45% (30/262) of the prasugrel-treated patients. On the basis of the pooled results, the incidence rate of HTPR in the prasugrel group was significantly higher than that in the ticagrelor group (RR = 0.30; 95% CI: 0.12–0.75; *P* = 0.01; Fig. 4b, Table 3). Furthermore, six studies reported LTPR based on VASP-PRI, including 60.68% of the ticagrelor-treated patients and 43.51% of the prasugrel-treated patients. The results of the pooled analysis showed a lower LTPR in the prasugrel group than in the ticagrelor group (RR = 1.40; 95% CI: 1.08–1.81; *P* = 0.01; Fig. 4c). Subsequently, a subgroup analysis based on study types, testing time, and special population was conducted. In the HTPR (PRU) group, the analysis results were not representative because only two studies were included. In the HTPR (PRI) group, a significant difference was observed in the RCT (WMD = 0.30; 95% CI: 0.12–0.75); *P* = 0.010) and < 24 h after the loading dose groups, which suggests the rate of HTPR was lower in the prasugrel group than in the ticagrelor group. For the rate of LTPR (PRI), a significant difference was found only at 5–30 days after treatment initiation (Table 3).

### Publication bias

Egger’s test was performed to evaluate the potential publication bias in the present study, and the results showed no significant bias was detected in the included studies [PR (PRU), *P* = 0.688; PR (PRI), *P* = 0.127; HTPR rates (PRU), *P* = NA (the Egger test could not be performed because only two studies were included); HTPR rates (PRI), *P* = 0.199; and LTPR rates (PRI), *P* = 0.243].

## Discussion

This meta-analysis of 14 studies compared the effects of ticagrelor and prasugrel on HTPR and LTPR according to VASP-PRI and PRU in patients with ACS. Our results showed that the ACS patients treated with prasugrel had a higher PR than those treated with ticagrelor. In addition, a lower HTPR based on VASP-PRI was found in the ACS patients treated with ticagrelor than in those treated with prasugrel. Furthermore, the results showed that a lower LTPR based on VASP-PRI was observed in the prasugrel group than in the ticagrelor group.

Ticagrelor is a cyclopentyl triazolopyrimidine that directly targets the P2Y12 platelet receptor [29]. As a novel P2Y12 blocker, ticagrelor is more potent and rapid-acting than clopidogrel. The randomized PLATelet inhibition and patient Outcomes (PLATO) study showed that ticagrelor reduced the incidence rates of cardiovascular death, myocardial infarction, and stroke compared with clopidogrel [6]. In addition, ticagrelor reduces the adenosine uptake by red blood cells, decreasing its plasma concentration, and inhibits platelet aggregation [30, 31]. Prasugrel is a third-generation thienopyridine, and its active metabolite irreversibly inhibits the P2Y12 receptor by blocking its binding site, thereby inhibiting ADP-induced platelet aggregation [32]. Similarly, prasugrel also shows a clinical advantage over clopidogrel. In the Trial to Assess Improvement in Therapeutic Outcomes by Optimizing Platelet Inhibition with Prasugrel-Thrombolysis in Myocardial Infarction 38 (TRITON-TIMI 38), prasugrel administration significantly reduced the rates of ischemic events [33]. Although both ticagrelor and prasugrel were outstanding antiplatelet agents, we revealed that prasugrel might enable a higher PR than ticagrelor. Alexopoulos et al. [7] indicated that ticagrelor produced a significantly higher platelet inhibition compared with prasugrel, which was consistent with our findings. This phenomenon was presumably explained by the fact that prasugrel irreversibly inhibited the P2Y12 receptor, whereas ticagrelor was a reversible P2Y12 blocker [34]. Moreover, the inhibition of adenosine uptake may promote the antiplatelet effects of ticagrelor. The Intracoronary Stenting and Antithrombotic Regimen: Rapid Early Action for Coronary Treatment (ISAR-REACT) 5 trial compared the impacts of ticagrelor and prasugrel on clinical events in 4000 patients [22], providing a reference based on the clinical point of view. This study suggested that ticagrelor showed a stronger antiplatelet effect than prasugrel, which was further supported our results. Unexpectedly, the study also found that the risk of myocardial infarction at 1 year after the ISAR-REACT 5 trial was significantly lower in the prasugrel group than in the ticagrelor group. Likewise, Deharo et al. [11] suggested that ticagrelor administration was associated with higher platelet inhibition and incidence of a hyper-response than prasugrel administration 1 month after ACS, which possibly exposes patients to a higher risk of bleeding complications. The assessment of end points or measurement timing might have caused this difference.

Different individuals had various responses to antiplatelet therapy, and the residual reactivity of platelets after antiplatelet therapy was also different. Thus, testing the residual reactivity might be one of the methods to evaluate the reactivity of patients to antiplatelet therapy and the risk of thrombosis. Therefore, we compared the effects of prasugrel and ticagrelor on HTPR and LTPR. Our results showed a significant difference in HTPR according to the VASP-PRI test between the ticagrelor- and prasugrel-treated groups. Notably, HTPR is influenced by different detection methods and evaluation criteria [35, 36]. However, only two studies reported PRU-based HTPR, and the results revealed no significant difference between the prasugrel and ticagrelor groups. Ferreiro et al. [37] reported that the HTPR assessed using VASP-PRI was higher than that assessed using PRU in prasugrel-treated patients. In addition, HTPR assessed using VASP-PRI is reported to be significantly lower after ticagrelor treatment than after prasugrel treatment [16]. Ticagrelor administration reduces the plasma concentration of adenosine and the activation of cyclic adenosine monophosphate-dependent protein kinases that inhibit VASP phosphorylation; however, it does not influence the PR calculated based on PRU [30, 31, 38]. The ISAR-REACT 5 trial [22] indicated that the incidence of myocardial infarction was significantly lower in prasugrel-treated patients with ACS than in ticagrelor-treated; and HTPR reduced the inhibition rate of platelet aggregation in vivo, leading to high platelet residual reactivity, which might have a higher risk of thrombotic events. In this study, we found prasugrel might enable higher PR than ticagrelor. However, the level of HTPR was lower in the patients with ACS treated with ticagrelor than in those treated with prasugrel. Our findings were inconsistent with the results of ISAR-REACT 5 trial, this inconsistency might be attributed to the testing methodand the sample size. Notably, HTPR is defined as a modifiable risk factor or only the marker of adverse reactions this event remaines controversial. Future studies should take this factor into account.

In addition, we found that in all the included studies, the LTPR rate was lower in the prasugrel group than in the ticagrelor group. LTPR reduced a high inhibition rate of platelet aggregation in vivo, resulting in an excessively low reactivity of platelet residues, which might lead to a higher blood risk. Observational studies have suggested an association between LTPR and bleeding, indicating that the occurrence of bleeding events in patients treated with P2Y12 receptor inhibitors is related to the excessive platelet inhibition with the consequence of LTPR [39]. Siller-Matula et al. [40] observed that 69% of the patients in the prasugrel group and 64% of the patients in ticagrelor group displayed LTPR. Another study corroborated these findings [41]. Unfortunately, our findings were inconsistent with previous studies. We proposed that the presence of confounding factors in the cohort studies might have exaggerated the difference between the two groups. Specifically, the thresholds for LTPR (VASP-PRI < 20% or < 16%), small size (only 4 RCTs), population characteristics (ACS patients with STEMI or diabetes), timing of measurements (1 month, 6–12 h, and other), and mean age (50, 60, or 70 years) might have confounded these findings.

This study has several limitations. First, the differences in cutoff values to define low or higher responders using VASP-PRI were not considered. Second, the study focused on PR as the primary end point and was insufficiently powered to compare clinical end points. Third, owing to the limited sample size, the subgroup analysis results were not discussed in depth. Fourth, the total number of pooled results for LTPR from the cohort studies was inconsistent with the number of pooled results from the RCTs. Therefore, additional RCTs should be examined to verify our results. Finally, ticagrelor could inhibit erythrocyte absorption of adenosine and subsequent increase in plasma adenosine concentration, which might activate A2 adenosine receptors on platelets, increase cAMP levels, and induce phosphorylation of camp-dependent protein kinases on VASP. Thus, the differences identified by VASP-PRI and PRU might be just methodological errors. These findings still need further elaboration.

## Conclusion

Prasugrel might allow a higher PR than ticagrelor. Ticagrelor could lead to a decreased HTPR and increased LTPR. Several uncertainties such as the nondeterminacy of the effectiveness of ticagrelor that was estimated with the VASP-PRI or the definition of HTPR (high or modifiable risk factor) might have affected our results.

## Data Availability

The datasets generated and analyzed during the present study are available from the corresponding author on reasonable request.

## Abbreviations

ACS: Acute coronary syndrome
CI: Confidence interval
HTPR: High on-treatment platelet reactivity
LTPR: Low on-treatment platelet reactivity
NSTEMI: Non-ST-segment elevation myocardial infarction
PCI: Percutaneous coronary intervention
PRU: P2Y12 reaction unit
RCT: Randomized controlled trial
RR: Risk ratio
STEMI: ST-segment elevation myocardial infarction
VASP-PRI: Vasodilator-stimulated phosphoprotein platelet reactivity index
WMD: Weighted mean difference
